# The Chromosome 19 microRNA Cluster Facilitates Cancer Stemness in Hepatocellular Carcinoma

**DOI:** 10.3390/ncrna11060074

**Published:** 2025-10-29

**Authors:** Marian T. Underwood, Varsha Devarapalli, Goodwin G. Jinesh, John H. Lockhart, Marco Napoli, Nino Mtchedlidze, Elsa R. Flores, Andrew S. Brohl

**Affiliations:** 1 Department of Molecular Oncology, H. Lee Moffitt Cancer Center & Research Institute, Tampa, FL 33612, USA; varsha.devarapalli@moffitt.org (V.D.); goodwinjinesh@gmail.com (G.G.J.); john.lockhart@moffitt.org (J.H.L.); marco.napoli@moffitt.org (M.N.); nmtchedlidze4680@eagle.fgcu.edu (N.M.); elsa.flores@moffitt.org (E.R.F.); 2 Molecular Medicine Program, H. Lee Moffitt Cancer Center & Research Institute, Tampa, FL 33612, USA; 3 Department of Immunology, H. Lee Moffitt Cancer Center & Research Institute, Tampa, FL 33612, USA; 4 Sarcoma Department, H. Lee Moffitt Cancer Center & Research Institute, Tampa, FL 33612, USA

**Keywords:** C19MC, stemness, HCC, CRISPR

## Abstract

**Background/Objectives:** Hepatocellular carcinoma (HCC) is one of the world’s deadliest cancers; however, the mechanisms that contribute to its aggressiveness are poorly understood. In the recent literature, overexpression of the Chromosome 19 MicroRNA Cluster (C19MC) has been associated with an aggressive phenotype and unfavorable prognosis in HCC. However, the molecular consequences of C19MC overexpression in HCC remain poorly understood. **Methods**: Here, we created a constitutive C19MC-overexpressing HCC model and used two different CRISPR-engineered C19MC-overexpressing HCC models to analyze phenotype and transcriptomic changes. **Results**: We observed that C19MC overexpression induces cancer stem cell (CSC) phenotypic features in vitro and analyzed transcriptomic changes in genes correlating with stemness, such as NFκB and EMT. **Conclusions**: C19MC induces changes in HCC that are consistent with stemness and aggression, which provides a better understanding of why C19MC could be a biomarker of poor prognosis.

## 1. Introduction

The Chromosome 19 MicroRNA Cluster (C19MC) is a maternally imprinted [1] primate-specific miRNA cluster located on the ‘q’ arm of human chromosome 19 [2]. It spans ~100 kb and contains 46 miRNAs expressed primarily in placental tissue [3], where it has been shown to act as a key regulator of trophoblast migration [3] and an innate antiviral response mechanism [4]. Recently, C19MC overexpression has also been implicated in a wide variety of cancer types, including undifferentiated embryonal sarcoma of the liver (UESL) [5], the embryonal tumor with multilayered rosettes (ETMR) [6,7], triple negative breast cancer [8], melanoma [9], and hepatocellular carcinomas [10,11]. Additionally, many of the microRNAs within C19MC have the AAGUGC seed motif, which has been linked with oncogenic signaling [12].

The literature supports a pathological role of C19MC overexpression in hepatocellular carcinoma (HCC) [13]. One study demonstrated that C19MC is frequently overexpressed in HCC tumors compared to normal liver tissue [14]. Additionally, C19MC expression levels have been demonstrated to have prognostic implications amongst HCC patients, with high expression correlating with poorer survival [13]. Several potential mechanisms for C19MC driving poor prognosis in HCC have been proposed. In human tumor specimens, C19MC overexpression is associated with a primitive and undifferentiated phenotype marked by high expression of cancer testis antigens [10,15]. In earlier studies by our group, we have shown that overexpression of individual C19MC miRNAs can induce multiple CSC-associated pathways in HCC cell lines, including the promotion of cancer testis antigen mRNAs [10], MYO18 mRNA dysregulation [11], and alternative meiotic nuclear division in human cancers [16].

C19MC miRNAs are thought to be expressed as a single cistron and be regulated at the cluster level, as the expression levels of individual miRNAs within the cluster exhibit high correlation. Therefore, to more closely model disease biology than previous models that overexpress only singular C19MC miRNAs [10,11], we generated HCC cell models that constitutively overexpress the cluster-wide C19MC using two CRISPR-based approaches. Using these in vitro models, we evaluated the phenotypic and transcriptional profile changes induced by overexpression of the whole C19MC cluster in human HCC.

## 2. Results

### 2.1. Generation of Two HCC Cell Lines That Constitutively Overexpress the Cluster-Wide C19MC miRNAs

**Figure 1:**
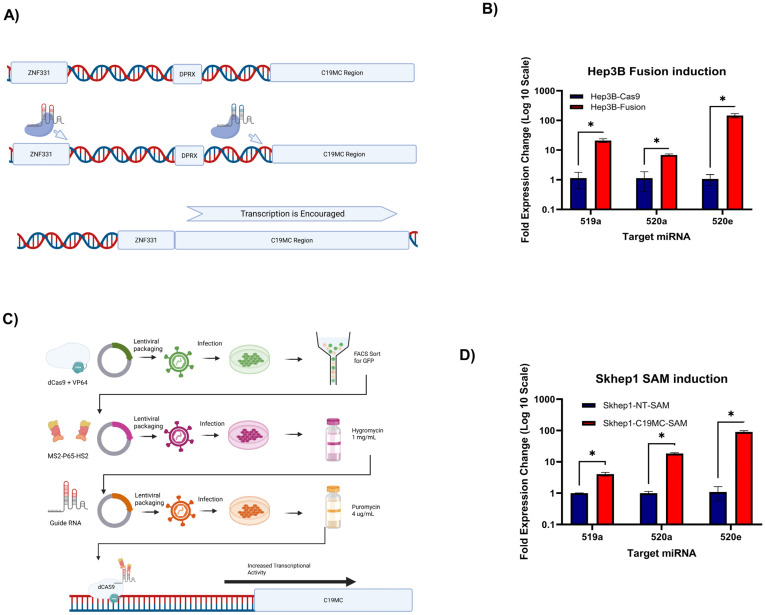
**Creation and verification of whole-cluster C19MC overexpression models using CRISPR.** (**A**) A schematic representation of the fusion event CRISPR-engineered in the Hep3B-ZNF331-C19MC-Fusion model. (**B**) Transcriptional levels of the three selected microRNAs to indicate cluster-wide overexpression in Hep3B (**C**). A schematic of the viral infection process for the creation of the Skhep1-C19MC-SAM model. (**D**) Transcriptional levels of the three selected microRNAs to indicate cluster-wide overexpression in Skhep1. Asterisk (*) indicates significance, which is defined as a *p*-value < 0.05.

To evaluate the effects of cluster-wide C19MC expression, we utilized two parallel CRISPR techniques to develop two different cell models. The first method mimics fusion-driven C19MC overexpression, which has been reported as a driver structural variant genomic change observed in several C19MC-overexpressing tumor types, such as UESL [5] and ETMR [17]. Using a CRISPR-SpCas9 vector [18], we induced an artificial fusion event between C19MC and 3’-UTR of ZNF331, a locus ~85 kb upstream of the C19MC start site in Hep3B cells. Hep3B has low endogenous expression levels of C19MC, but when fused to the 3’-UTR of transcriptionally active ZNF331, the result is cluster-wide C19MC overexpression (Figure 1A). We verified the fusion, as well as marked overexpression of representative microRNAs as described in our previous work [19].

For a second method to induce cluster-wide C19MC overexpression, we utilized the CRISPR-based Synergistic Activation Mediator (SAM) methodology [20] to create the Skhep1-C19MC-SAM model and its paired nontargeted control, Skhep1-NT-SAM (Figure 1B). The SAM Methodology utilizes a ‘decatalyzed’ Cas9 protein to anchor a set of transcriptional activators to the location of the designed CRISPR guide, which rapidly encourages transcription [20]. We designed the guide RNA to target the TATA-box location at the C19MC start site for this model. The expression of the C19MC miRNAs across the cluster was evaluated using the same TaqMan qRT-PCR strategy as the Hep3B models, and the levels of overexpression were like those of our ZNF331-C19MC fusion model (Figure 1C,D).

### 2.2. C19MC Overexpression in HCC Cell Lines Promotes Upregulation of Stemness-Related Genes and Gene Sets

**Figure 2:**
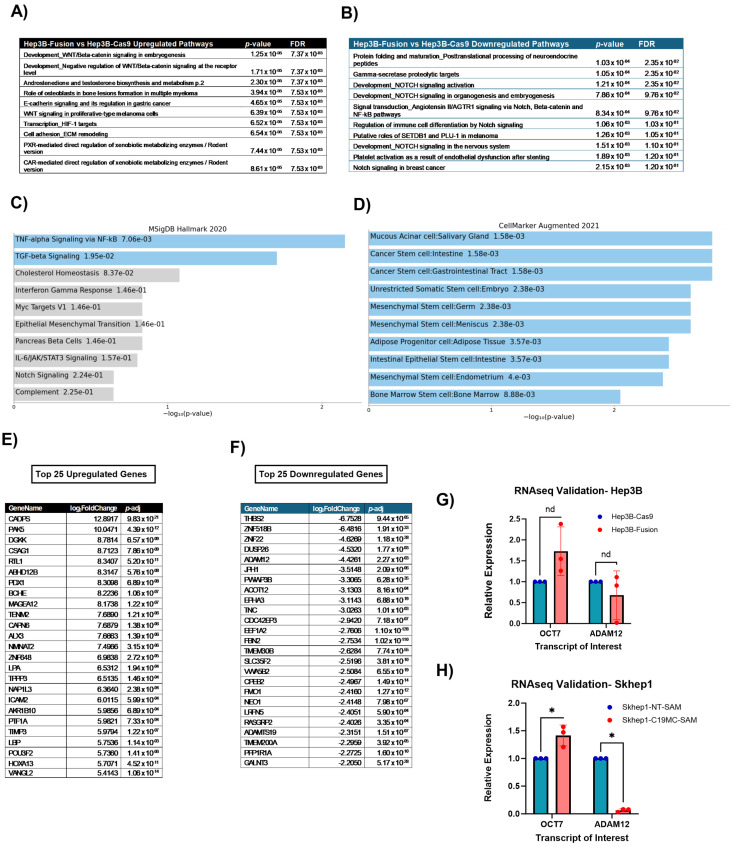
**C19MC-overexpressing models demonstrate signatures consistent with cancer stem cells.** (**A**) Significant upregulated pathways as defined by MetaCore Analysis. (**B**) Significant downregulated pathways as defined by MetaCore Analysis. (**C**) Hallmark results of C19MC target site-enriched genes. (**D**) CellMarker results of C19MC target site-enriched genes. (**E**) Significantly upregulated genes organized by differential expression. (**F**) Significantly downregulated genes organized by differential expression. (**G**) Transcriptional validation of selected target genes in the Hep3B-Fusion model by SYBR green qRT-PCR. (**H**) Transcriptional confirmation of the Hep3B differentially expressed target genes in the Skhep1-C19MC-SAM model by SYBR green qRT-PCR. Asterisk (*) indicates significance, which is defined as a *p*-value < 0.05. The symbol “nd” indicates insignificance, or a *p*-value > 0.05.

To look at potential cancer-causing pathways that are altered by C19MC overexpression, we performed RNA sequencing on our Hep3B-ZNF331-C19MC-Fusion (referred to as ‘Hep3B-Fusion’) and the Hep3B-SpCas9 (referred to as ‘Hep3B-Cas9’) control cell lines. A MetaCore Analysis comparing gene expression in Hep3B-Fusion to Hep3B-Cas9 control cells revealed the upregulation of multiple pathways associated with cell stemness, such as ‘Development_WNT/Beta-catenin signaling in embryogenesis’ [21] and ‘Transcription of HIF-1 targets’ [22] (Figure 2A), as well as repression of pathways responsible for cellular differentiation (Figure 2B) supporting a causative link between C19MC cluster-wide overexpression and a shift towards a cancer stem cell (CSC)-like transcriptional profile. Notably, the ‘Development_NOTCH’ signaling activation was identified as a downregulated process in the Hep3B-Fusion model. The Notch pathway, although context dependent, is a key regulator of cell fate decisions, and the aberration of it often results in cytotoxic affects to the cell [23]. To evaluate for potential direct C19MC miRNAbinding sites to stemness-related genes, we utilized TargetScan, a microRNA binding site prediction tool. Using TargetScan [24,25], we identified putative direct gene targets for all 46 of the C19MC microRNAs. In total, there were 1110 binding sites in 1023 unique genes with high-confidence predicted 3′ UTR binding sites for at least one of the C19MC miRNAs. We first compared the predicted C19MC miRNA target genes to the list of differentially expressed genes identified by RNAseq data. Ten genes of interest from the TargetScan data overlapped with the 173 upregulated genes of interest from RNAseq (*p* < 0.05, log_2_Foldchange > 2.0) *AGTR2,ABEC3C,DUSP5,LPA,LYZ,NPIL3,SEMA5B,SH3TC2,ST3GAL1,TM4SF1*. Additionally, only 3 of 34 significantly downregulated genes (*p* < 0.05, log_2_Fold change < −2.0) overlapped with the TargetScan predicted miRNA targets: *FUT1, GALNT3,* and *ZNF22*. This suggests that most of the global gene expression changes that result from C19MC overexpression are not due to direct interaction but are instead from downstream effects. Therefore, we additionally performed pathway analysis of the TargetScan-predicted C19MC miRNA gene targets. Interestingly, when compared to MSigDB Hallmark gene sets, there was significant enrichment for genes in the TGF-β and NFκB signaling pathways (Figure 2C). Additionally, when compared to CellMarker Augmented gene sets, there was significant enrichment for multiple stem cell signatures (Figure 2D). Taken in whole, predicted C19MC miRNA gene targets are enriched for stemness-related gene pathways, congruent to RNAseq differential expression analysis, despite limited direct overlap between differentially expressed genes and predicted direct miRNA gene targets. Based on the MetaCore and the TargetScan analyses, we observed stemness- and differentiation-related genes to be amongst the most differentially expressed in this model (Figure 2E,F). Notable upregulated genes with strong literature ties to stemness included HOXA1 [26], PAK5 [27], and OCT7 (also known as POU3F2) [28], and downregulated genes linked to cellular differentiation included ADAM12 [29] and NEO1 [30]. Finally, to confirm our sequencing results and evaluate the consistency of expressional changes across both cell line models, we performed qRT-PCR on selected stemness-related genes. We observed significant overexpression of POU3F2 and repression of ADAM12 in both the Hep3B-Fusion (Figure 2G) and Skhep1-C19MC-SAM cell lines (Figure 2H), compared to their respective controls. Taken together, we observed that cluster-wide C19MC overexpression leads to global transcriptional changes related to stemness and differentiation features, including both at the gene set and individual gene level, and that the transcriptional changes were consistently observed across both of our C19MC overexpression models.

### 2.3. C19MC Overexpression in HCC Models Regulates the mRNA Expression of Stemness-Linked Cytokines (IL6, IL8, and IL1B) and Stemness-Related Transcription Factors

**Figure 3:**
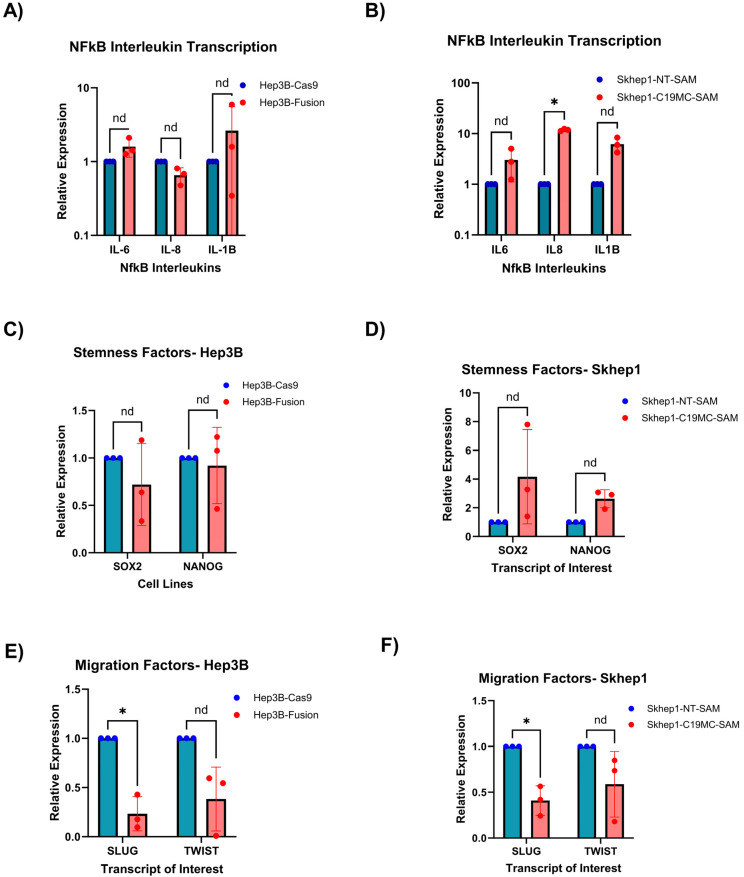
**C19MC overexpression in HCC alters key CSC-regulatory genes.** (**A**,**B**) Transcriptomic analysis of key NFkB, interleukin transcripts, quantified using SYBR green qRT-PCR at 48 hrs. Each data point represents 1 biological replicate for a total of *n* = 3 replicates. Asterisk (*) indicates significance, which is defined as a *p*-value < 0.05. The symbol “nd” indicates insignificance, or a *p*-value > 0.05. (**C**,**D**) Transcriptomic analysis of stemness factors Sox2 and Nanog, quantified using SYBR green qRT-PCR at 48 hrs. Each data point represents 1 biological replicate for a total of *n* = 3 replicates. (**E**,**F**) Transcriptomic analysis of iPSC-correlated migration factors, quantified using SYBR green qRT-PCR at 48 h. Each data point represents 1 biological replicate for a total of *n* = 3 replicates. Asterisk (*) indicates significance, which is defined as a *p*-value < 0.05. The symbol “nd” indicates insignificance, or a *p*-value > 0.05.

Inflammation plays a significant role in the development and progression of HCC [31] and in stem cell development and differentiation [32]. We therefore evaluated the transcriptional levels of proinflammatory cytokines IL-6, IL-8, and IL-1B as potential mediators of stemness in our C19MC-overexpressing models. These three cytokines are transcribed as part of the NFkB pathway to facilitate the inflammatory processes in the body [31,32,33]. Additionally, these three cytokines have been linked to the facilitation of stemness induction in cells through encouragement of plasticity and enrichment of CSC populations [34,35,36]. Here we observe that, out of the three cytokines tested, the Hep3B model trended toward upregulation of two of the three (IL-6 and IL-1B), with a minor reduction in levels of IL-8 (Figure 3A). In our Skhep1-C19MC-SAM model, however, we identified upregulation of all three cytokine transcripts, with IL-8 being significantly upregulated (Figure 3B).

To further evaluate potential mediators of stemness in our C19MC-overexpressing HCC cell lines, we measured the expression changes in canonical CSC transcription factors NANOG and SOX2 [37,38]. We observed a minor repression of these transcription factors in the Hep3B-Fusion model (Figure 3C) but an upregulation in the Skhep1-C19MC-SAM model (Figure 3D). We additionally evaluated transcriptional levels of SNAI2 (also known as SLUG) and TWIST, which are transcription factors governing the epithelial-to-mesenchymal transition (EMT) that have been shown to be repressed by C19MC expression in induced pluripotent stem cells (iPSCs) [39]. In both our Hep3B-Fusion and Skhep1-C19MC-SAM models, C19MC overexpression led to repression of transcriptional levels of SNAI2 and TWIST (Figure 3E,F). Taken as a whole, C19MC overexpression results in transcriptional changes in several potential mediators of stemness, including upregulation of proinflammatory and pro-stemness cytokines and repression of stemness-related transcriptional factors.

### 2.4. C19MC-Overexpressing HCC Models Exhibit Enhanced Cellular Fitness in Anchorage-Independent Growth Conditions

**Figure 4:**
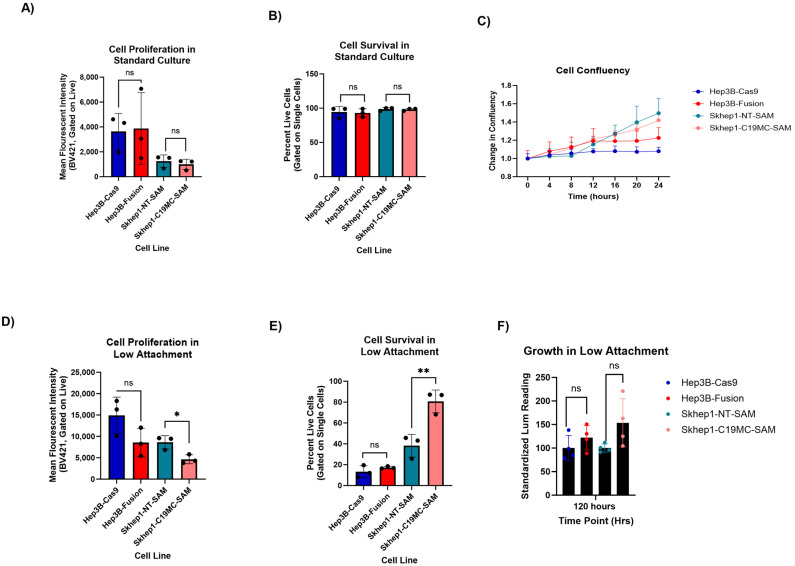
**C19MC-overexpressed HCC models have increased viability and increased proliferation under low-attachment growth conditions.** (**A**,**B**) CellTrace Violet signal intensity (**A**) and cell viability (**B**) after 5 days of growth in traditional 2D culture conditions in both HCC models. Each data point represents 1 biological replicate for a total of *n* = 3 replicates. Asterisk (*) indicates significance, which is defined as a *p*-value < 0.05. The symbol “nd” indicates insignificance, or a *p*-value > 0.05. (**C**) Change in confluency measured via the Incucyte Confluency module across 24 h. Data is representative of three biological replicates compiled into one data point with error bars representing the 95th percentile confidence interval. (**D**,**E**) CellTrace Violet signal intensity (**D**) and cell viability (**E**) across 5 days in low-attachment conditions for both HCC models. Each data point represents 1 biological replicate for a total of *n* = 3 replicates. Asterisk (* or **) indicates significance, which is defined as a *p*-value < 0.05. The symbol “ns” indicates insignificance, or a *p*-value > 0.05. (**F**) Cell viability was measured using the CellTiter Glo reagent (Luminescent ATP) at 5 days in low-attachment conditions. Each data point represents 1 biological replicate for a total of *n* = 4 replicates.

We next sought to assess the phenotypic changes caused by cluster-wide C19MC overexpression in our models. Given the observed link between C19MC overexpression and several stemness-associated gene sets, we evaluated cell line growth characteristics in both traditional 2D culture and in anchorage-independent growth conditions, as fitness advantage in the latter is a trait of CSC-like cells [40]. For anchorage-independent growth experiments, we utilized ‘growth in low attachment’ (GILA) assays [41]. As expected, C19MC overexpression did not lead to significant differences in cell proliferation, survival, or confluency in standard 2D culture (Figure 4A–C). However, our C19MC-overexpressing models were much more proliferative when cultured in the anchorage-independent growth conditions (Figure 4D). This is identified by the decrease in fluorescent intensity because the dye is depleted with each proliferative cycle. Additionally, C19MC-overexpressing models were much more fit in anchorage-independent conditions, due to their higher survival percentage compared to their respective control cell lines (Figure 4E). This was consistent with the results of the GILA assay, where the C19MC-overexpressing cell models produced more luminescent ATP in low-attachment conditions than their respective controls, which is a readout associated with metabolically fit and proliferating cells (Figure 4F). Overall, these data suggest that C19MC overexpression can create a more aggressive phenotype that is more likely to survive in stressful environments.

## 3. Discussion

The Chromosome 19 microRNA Cluster has been proposed as an Oncomir in multiple cancer types and a diagnostic and predictive biomarker in hepatocellular carcinoma [13,14,15]. We utilized two different CRISPR/Cas9 technologies to induce stable overexpression of the C19MC miRNAs in a cluster-wide fashion in HCC cell lines to better elucidate the global transcriptional changes that result from C19MC overexpression. We identified stemness-related pathways to be strongly altered by C19MC overexpression in our models and further highlighted key potential mediators of cellular stemness, including transcription factors and proinflammatory cytokines.

C19MC overexpression in our HCC cell lines led to molecular profiling changes often seen in cancer stem cells. Upregulation of WNT signaling and downregulation of Notch signaling are traits seen in the prevention of lineage differentiation [20,21]. The downregulation of Notch signaling is of exceptionally important note, as Notch regulation has been termed as the gatekeeping mechanism of cell fate decisions and, when downregulated, preserves the undifferentiated state of the cell [42]. Additionally, emerging work demonstrates the importance of Notch not just in control of the stem cell itself but rather the control of the niche around it, as the niche helps to determine the ultimate lineage decisions for the cell to fulfill [43]. Therefore, the downregulation of Notch signaling in response to C19MC overexpression demonstrates a potential mechanism of control over cell lineage decisions within the tumor niche, and further work will contribute to our understanding of how Notch and C19MC interact.

Inflammation has always been an integral part of the HCC disease biology, and the expression of IL-6 [44], IL-8 [35], and IL-1B [36] have all been shown to promote cancer cell stemness by reducing self-renewal pathways. SOX2 and NANOG transcriptional activity are key for the maintenance of pluripotency [36,37]. Our models demonstrate that C19MC induction leads to heightened mRNA expression of these key transcription factors and cytokines. The previous literature studying C19MC overexpression in an induced pluripotent stem cell model (iPSC) demonstrated repression of epithelial-to-mesenchymal transition and increased stemness markers when C19MC was overexpressed [39].

From a phenotypic standpoint, C19MC overexpression did not lead to notable changes in common cell line growth parameters when cultured in standard conditions. In contrast, C19MC-overexpressing cells exhibited significantly heightened cellular fitness when cultured in low-attachment conditions. The ability to survive and persist in stressful conditions is a trait used as a surrogate for malignancy, and malignant traits such as the preservation of tumor growth are often associated with CSCs [45]. Taken as a whole, our phenotypic results suggest that C19MC-induced oncogenic fitness may be specific to the maintenance of CSC-like cells, where the survival advantage may be more apparent in more tumor-like conditions.

A strength of our study is that we utilized multiple methodologies to induce expression of the C19MC miRNAs in a cluster-wide fashion. As any model system will have inherent artificiality, consistent results from orthogonal methods help to reduce validity concerns. Further, the CRISPRa model described in this report and ZNF331-C19MC-fusion model reported previously [19] are the first to induce cluster-wide overexpression of C19MC in HCC cells. Therefore, cluster-wide C19MC miRNA induction differs from previous studies that overexpress selected microRNAs from C19MC [10,11]. Our models provide insight into the effects of the overexpression of cluster-wide C19MC, as opposed to individual miRNAs. This more closely mirrors the observed expression pattern of the C19MC miRNAs in human HCC tumor samples [13].

However, one limitation of using two different cell models is some minor inconsistency between results, as the models are made using very different methodologies. The fusion model is made through a deletion of a ~85 kb region which consists of a silent gene *DPRX* and few silent ncRNA genes in the Hep3B cell line. Additionally, this model arose from a single cell clone. Our Skhep1-SAM model is made using a method that does not involve deletion and is polyclonal, which preserves the integrity of the genome but introduces cellular variance through p65 non-specific binding to genomes other than the dCas9-directed site, which should be nullified by the control and the number of cells in the pool. Due to the differences in cell lines, and model development, there might be small variances in gene expression results. This will be mitigated in future studies through the creation of more models utilizing the CRISPRa methodology, as this method has proven to be efficient and less time-intensive than the fusion–induction methodology.

In summary, we showed that C19MC cluster-wide overexpression in multiple HCC cell lines leads to global expression changes related to increased cellular stemness and that C19MC-overexpressing cells exhibit stem-like phenotypic growth characteristics. Future experiments are warranted to extend these findings, identify direct and indirect downstream targets of the C19MC miRNAs, and more conclusively link C19MC induction of stemness pathways to the poorer clinical outcomes observed in C19MC-overexpressing HCC cases. Our work could further present C19MC as a potential biomarker [13] in clinical settings to predict prognosis and outcome for patients receiving treatment.

## 4. Materials and Methods

### 4.1. Cell Culture

Skhep1 (ATCC, HTB-52), Hep3B (ATCC, HB-8064), and HEK293T (ATCC, CRL-11268) cell lines were maintained in Minimum Essential Medium (MEM) (Sigma-Aldrich, Darmstadt, Germany, 4655) with 10% FBS (Sigma-Aldrich, SIAL-F0926) and supplemented with L-glutamine (Gibco, Waltham, MA, USA, A2916801), vitamins (Gibco, 11120052), NEAA mix (Sigma-Aldrich, M7145), and Pen-Strep Solution (Gibco, 15140122). Cells were passaged 1–2 times a week with Trypsin-EDTA solution (Sigma-Aldrich, SIAL-T4049) at a ratio of (1:10), (1:4), and (1:10). All cell lines were verified using STR DNA fingerprinting and were routinely checked for mycoplasma contamination.

### 4.2. CRISPRa sgRNA Cloning

The sgRNA guide for the C19MC start site was cloned into the lenti sgRNA(MS2)_puro backbone (Addgene #73795, a gift from Feng Zhang) using the recommended Golden-Gate cloning protocol [19]. The sequence of the sgRNA guide used was (F: GAAATGCATTTACAGAAGCTAGG, R: CCTAGCTTCTGTAAATGCATTTC), which correlates with the TATA Box location upstream of the C19MC start site (hg38: 53664825-53664847). The product plasmid was then transformed into Stbl3 competent *E*. *coli* cells (Invitrogen, Waltham, MA, USA, C737303), and colonies were selected on 100 µg/mL Ampicillin-LB agar plates. Colonies for the plasmid were screened by PCR with flanking primers; PCR product bands were purified using the GFX gel purification kit (Cytiva, Marlborough, MA, USA 28-9034-70z0) and sequenced using Sanger Sequencing (Azenta, Burlington, MA, USA). Once the cloning of oligos was verified, the bacterial colony was inoculated into LB Broth with 100 µg/mL ampicillin overnight and then the plasmids were purified using the Qiagen Plasmid Isolation kit (Qiagen, Hilden, Germany, 12143).

### 4.3. CRISPR Methods

Hep3B-ZNF331-C19MC-Fusion and Hep3B-Cas9 control cells were described previously [18]. Skhep1-NT-SAM and Skhep1-C19MC-SAM cells were produced by sequential lentiviral infection of the dCAS9-VP64_GFP (Addgene #61422, a gift from Feng Zhang), lentiMPH V2 (Addgene #89308), and then either the C19MC guide (Skhep1-C19MC-SAM) or empty vector control (Skhep1-NT-SAM) were used. Cells were sorted for GFP using ARIA SORPII Cell Sorter after the first infection, treated with 500 µg/mL Hygromycin after the second infection, and after the final infection, cells were selected in 4 µg/mL Puromycin. Cells were maintained in 2 µg/mL Puromycin for the duration of the experiments.

### 4.4. Lentivirus Generation and Infection

Lentiviruses for each CRISPR-SAM component (dCas9-VP64, MS2-P65-HSF1, and the sgRNA-2xMS2aptemer) were created by transfecting psPAX2 (Addgene #12260, a gift from Didier Trono), Pmd2.G (Addgene #12259, a gift from Didier Trono), and the CRISPR-SAM component plasmid at a 1:1:3 ratio into 100,000 HEK 293T cells in a 6-well plate with Lipofectamine 2000 (Invitrogen, 11668500), as per manufacturer’s instructions. At a time of 48 h later, the supernatant was collected, briefly spun down to remove debris, and filtered through a 0.45 µm sterile filter. Supernatants were stored at −80 °C until use. Before administration to cells, 10% FBS and 10 µg/mL polybrene were added to the supernatant to encourage infection. Cells were incubated for 48 h after transfection before the media was changed to fresh culture media with selection agents.

### 4.5. MicroRNA Expression Analysis

RNA was collected from the cell lines using the Qiagen MicroRNAeasy kit (Qiagen, 217004) per the manufacturer’s instructions. The isolated RNA was then converted into small RNA-enriched cDNA using the Invitrogen High-Capacity Reverse Transcription Kit (Invitrogen, 4368814) and synthesis primers for U18 (4427975, Assay ID: 001204), miR-519a (4427975, Assay ID: 002415), miR-520E (4427975, Assay ID: 001119), and miR-520a (4427975, Assay ID: 001167) per the manufacturer’s instructions. Then, TaqMan master mix was prepared with TaqMan probes for U18, miR-519a, miR-520E, and miR-520a, respectively. Samples were analyzed by qRT-PCR on the QuantStudio 6 in triplicate.

### 4.6. RNA Sequencing and Analysis

RNA sequencing of Hep3B-ZNF331-C19MC-fusion and Cas9 control cells were described previously [19]. Results were matched to reference genome hg38 and quality controlled using RSEM 1.3.0. Pathway analysis was performed using MetaCore Bioinformatics Suite (Clarivate, London, UK). Differentially expressed upregulated genes were defined as genes that had log_2_fold change values > 2.0 and were significant (*p* < 0.05), and differentially expressed downregulated genes were defined as genes that had log_2_fold change values <−2.0 and were significant (*p* < 0.05).

### 4.7. TargetScan Enrichment and Analysis

Using TargetScan 8.0 [24,25], we generated gene target results for all of the 46 C19MC microRNAs except for hsa-miR-526, which does not have its own annotation in the TargetScan database but instead shares and annotation with hsa-miR-519, since the seed sequences are the same. To define ‘high-confidence’ for target site prediction, we utilized a cut of for cumulative weighted context++ score of <−0.4. From the cumulative findings, we condensed to the gene level for gene set enrichment analysis using EnrichR [46,47,48]. We utilized the ‘MSigDB_Hallmark_2020’ and ‘CellMarker_2024’ libraries to generate our final output.

### 4.8. CellTrace Violet Assay

A total of 500,000 cells were stained with CellTrace Violet dye (Invitrogen, C34557) per the manufacturer’s instructions. A sample of the stained and unstained cells was fixed immediately in 4% formaldehyde for baseline measurements. For low-attachment cultures, 100,000 stained cells were plated in each well of a 6-well Ultra Low Attachment plate (Corning, Corning, NY, USA, CLS3471). For normal 2D cultures, 50,000 stained cells were plated per standard 10 cm TC-treated dish. Both cultures were incubated for 5 days and then collected and stained with Zombie NIR live/dead dye (Biolegend, San Diego, CA, USA, 423105) per the manufacturer’s instructions. The 5-day time point was selected based on a comparison of results at the 3-day time point. Samples were briefly fixed in 4% formaldehyde and then analyzed by flow cytometry on a BD FACSCelesta to assess proliferation.

### 4.9. mRNA Expression Analysis

A total of 50,000 cells were plated in a 6-well plate. RNA was isolated 48 h later using the Qiagen miRNAeasy kit and quantified using a Nanodrop. The time point of collection was based on titration of 24 h, 48 h, and 72 h. cDNA was made from 0.5 to 1 µg of RNA (samples being compared were reverse transcribed with equivalent amounts of RNA), using the QuantaBio SuperScript 5X cDNA master mix (Quantabio, Beverly, MA, USA, 76533-174) per the manufacturer’s protocol. cDNA was diluted at 1:5, combined with respective primers and Quantabio SYBR Green Fast mix (Quantabio, 101414-292), and analyzed on a QuantStudio 6. All samples were analyzed in triplicate with three biological replicates for each cell line. Primers were added as per the Quantabio SYBR Green Fast mix’s protocol were used in pairs as follows: B-Actin (F: CATGTACGTTGCTATCCAGGC, R: CTCCTTAATGTCACGCACGAT); POU3F2(OCT7) (F: GTGTTCTCGCAGACCACCATCT, R: GCTGCGATCTTGTCTATGCTCG); ADAM12 (F: ATGGCATCTGCCAGACTCACGA, R: GGAA-CTCTTCGAGACTTTGCCAC); IL-6 (F: AGACAGCCACTCACCTCTTCAG, R: TTCTGCCAGTGCCTCTTTGCTG); IL-8 (F: GAGAGTGATTGAGAGTGGACCAC, R: CACAACCCTCTGCACCCAGTTT); IL-1B (F: CCACAGACCTTCCAGGAGAATG, R: GTGCAGTTCAGTGATCGTACAGG); SOX2 (F: GAGCTTTGCAGGAAGTTTGC, R: GCAAGAAGCCTCTCCTTGAA); NANOG (F: GGTGGCAGAAAAACAACTGG, R: CATCCCTGGTGGTAGGAAGA); SNAI2 (F: TGTTGCAGTGAGGGCAAGAA, R: GACCCTGGTTGCTTCAA-GGA); TWIST (F: GGAGTCCGCAGTCTTACGAG, R: TCTGGAGGACCTGGTAGAGG). POU3F2 and ADAM12 primers were purchased from Origene, while all others were synthesized from Millipore-Sigma.

### 4.10. Growth in Low Attachment (GILA) Assay

GILA was prepared as described [41]. Each cell line was plated at 5000 cells per well in a flat-bottom, low-attachment 96-well plate and incubated for 5 days. At the designated time point, cell viability was measured using CellTiter Glo (Promega, Madison, WI, USA, G7570) per the manufacturer’s instructions. Lysates were moved to a black-bottomed 96-well plate and then assayed for luminescence on a Biotek Synergy HS1 microplate reader. Samples were assayed in triplicate and with five biological replicates for each cell line.

### 4.11. Cell Confluency Assays

A total of 5000 cells were plated per well in a standard 96-well plate and left to adhere for 16 h. Plates were moved to an Incucyte SX5 (Sartorius, Göttingen, Germany), and confluency was measured every 6 h for three days using the standard Confluency module. Cell lines were analyzed in triplicate with a total of three biological replicates.

### 4.12. Statistical Analysis

Statistical analysis for the MetaCore, EnrichR, and TargetScan analyses were calculated automatically by each program, respectively, and cutoffs were determined as described previously in each methods section describing the analysis. Statistical analysis of qRT-PCR, flow cytometry, Incucyte, and GILA data was performed using GraphPad Prism Version 10. qRT-PCR, flow cytometry, and GILA data were interpreted using an unpaired *t*-test, with *p* < 0.05 signifying significance. Linear regression analysis for the Incucyte Cell Confluency data was performed using the Linear Regression Analysis comparison within the GraphPad Prism Statistics suite.

## 5. Patents

The authors G.G.J. and A.S.B. have a provisional patent application pending for the CRISPR-ZNF331-C19MC-fusion guide RNAs and the Hep3B-Fusion cells engineered with these guides.

## Data Availability

The raw data supporting the conclusions of this article will be made available by the authors on request.

## Abbreviations

The following abbreviations are used in this manuscript:

C19MC	Chromosome 19 microRNA Cluster
CSC	Cancer stem cell
HCC	Hepatocellular carcinoma
